# Diagnosis of Uncomplicated and Complicated Appendicitis in Adults

**DOI:** 10.1177/14574969211008330

**Published:** 2021-04-14

**Authors:** W. J. Bom, J. C. G. Scheijmans, P. Salminen, M. A. Boermeester

**Affiliations:** 1 Department of Surgery, Amsterdam UMC, location AMC, Amsterdam Gastroenterology Endocrinology Metabolism, University of Amsterdam, Amsterdam, The Netherlands; 2 Department of Surgery, University of Turku, Division of Digestive Surgery and Urology, Turku University Hospital, Turku, Finland

**Keywords:** Acute appendicitis, imaging, adults, complicated appendicitis

## Abstract

**Background:**

Diagnostic work-up of acute appendicitis remains challenging. While some guidelines advise to use a risk stratification based on clinical parameters, others use standard imaging in all patients. As non-operative management of uncomplicated appendicitis has been identified as feasible and safe, differentiation between uncomplicated and complicated appendicitis is of paramount importance. We reviewed the literature to describe the optimal strategy for diagnosis of acute appendicitis.

**Methods:**

A narrative review about the diagnosis of acute appendicitis in adult patients was conducted. Both diagnostic strategies and goals were analyzed.

**Results:**

For diagnosing acute appendicitis, both ruling in and ruling out the disease are important. Clinical and laboratory findings individually do not suffice, but when combined in a diagnostic score, a better risk prediction can be made for having acute appendicitis. However, for accurate diagnosis imaging seems obligatory in patients suspected for acute appendicitis. Scoring systems combining clinical and imaging features may differentiate between uncomplicated and complicated appendicitis and may enable ruling out complicated appendicitis. Within conservatively treated patients with uncomplicated appendicitis, predictive factors for non-responsiveness to antibiotics and recurrence of appendicitis need to be defined in order to optimize treatment outcomes.

**Conclusion:**

Standard imaging increases the diagnostic power for both ruling in and ruling out acute appendicitis. Incorporating imaging features in clinical scoring models may provide better differentiation between uncomplicated and complicated appendicitis. Optimizing patient selection for antibiotic treatment of appendicitis may minimize recurrence rates, resulting in better treatment outcomes.

## Acute Appendicitis

Appendicitis is the most common infectious disease in the abdomen. With a lifetime risk of almost 1 in 11 persons, appendicitis has been diagnosed in innumerable patients worldwide (1). Still, there is a lot to learn about the diagnostic approach. Guidelines vary in their advice for standard diagnostics (2,3). Multiple clinical prediction rules have been described during the past decades (4). Most scores provide some evidence for a risk stratification without including imaging features. For practicing such clinical scores, selective imaging has been proposed; a score result in the low-risk category may end further investigation for the diagnosis of acute appendicitis, an intermediate risk score may lead to imaging, and a high-risk score may result in direct surgical exploration (3). While some guidelines advise the use of clinical scoring systems, others recommend standard imaging in all patients with suspected appendicitis (5).

Besides reliable diagnosis of acute appendicitis instead of alternative explanations of abdominal pain, discriminating uncomplicated from complicated appendicitis becomes more and more relevant as evidence is growing for the feasibility of treatment with antibiotics compared to surgery in uncomplicated appendicitis (6,7). This discrimination is based on the principle that uncomplicated and complicated appendicitis are two different entities (8–10). Simple or uncomplicated appendicitis is defined as a phlegmonous inflamed appendix without signs of necrosis or perforation, whereas complex or complicated appendicitis has focal or transmural necrosis, which eventually may lead to perforation. Differentiation between both entities is important, as uncomplicated appendicitis may be treated conservatively with antibiotics without the need for surgery (6,7), or may even resolve spontaneously without the need for antibiotic treatment (9,11,12). In contrast, patients with complicated appendicitis require emergency appendectomy with the exception of patients presenting with a periappendicular abscess (3,13).

In this narrative review, we will focus on the different ways of diagnosing acute appendicitis, discuss the considerations, and zoom in on the differentiation between uncomplicated and complicated appendicitis. We will base our view on available literature, preferring the use of randomized controlled trials or well-designed meta-analyses over single cohort studies.

## Diagnostic Accuracy: To Rule In or to Rule Out?

**Fig. 1.**
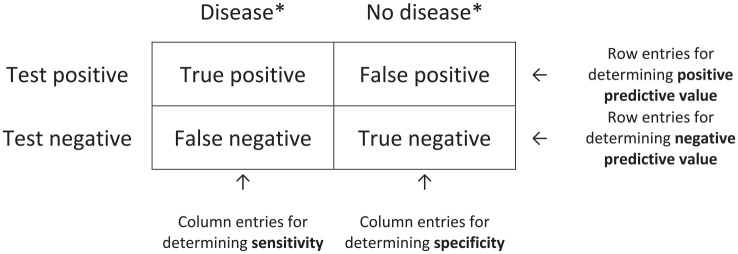
Standard contingency table. *Status of person according to “Gold Standard.”

When diagnosing acute appendicitis in a patient with abdominal pain, the diagnostic accuracy is, in a simplified way, based on the classical contingency or 2 × 2 table, see Fig. 1.

However, the actual diagnostic situation is more complex. Patients with abdominal pain and suspicion of appendicitis present with a spectrum of diseases, including the two levels of appendicitis severity. The difficult diagnostic task is to differentiate between at least four different categories:

1. Patients with abdominal pain without appendicitis and with no other condition needing treatment (traditionally called non-specific abdominal pain (NSAP)) and therefore do not need to be diagnosed or treated.
2. Patients with acute appendicitis:
  (a) Patients with uncomplicated appendicitis who do not need urgent surgical treatment or surgical treatment at all.
  (b) Patients with complicated appendicitis in need of urgent surgical treatment.
3. Patients with other conditions who need further diagnostic work-up or treatment.

**Fig. 2.**
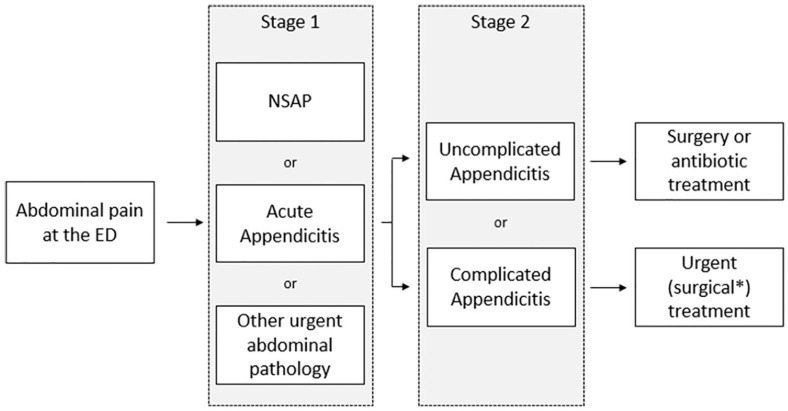
“Two-stage” diagnostic approach. ED: emergency department; NSAP: non-specific abdominal pain. *Except for patients with an intra-abdominal abscess.

To diagnose acute appendicitis correctly, a “two-stage” approach is suggested, see Fig. 2. In the first stage, the diagnosis “acute appendicitis” needs to be made. For patients without acute appendicitis, a correct other cause of their complaints needs to be found, as some abdominal pathologies require urgent treatment. After confirming the diagnosis of acute appendicitis, a distinction will be made between uncomplicated and complicated appendicitis in the second stage, as different treatment options can be considered for these different diseases.

There are several strategies to diagnose the different categories of abdominal pathology in stage 1. For selecting the right diagnostic approach, it is important to clarify the diagnostic goal: ruling in or ruling out a disease. To rule in a disease, both specificity and positive predictive value (PPV) need to be high, while for ruling out, sensitivity and negative predictive value (NPV) should both be high. At higher sensitivity, specificity may be lower, and vice versa. Diagnostic characteristics need to be considered when selecting a diagnostic test.

In case of diagnosing acute appendicitis in the first diagnostic stage, low sensitivity or low NPV may lead to discharge from the emergency room (ER) of patients who actually have appendicitis. Missed diagnoses lead to treatment delay. In patients with uncomplicated appendicitis, a delay for up to at least 24 h does not appear to increase the postoperative complication rate (13). However, in complicated appendicitis, delaying appendectomy leads to more complications (13). In contrast, low specificity or low PPV may lead to overdiagnosis, causing high negative appendectomy rates (NARs). Therefore, both ruling in and ruling out acute appendicitis are important.

For discrimination between uncomplicated and complicated appendicitis in the second diagnostic stage, ruling out complicated appendicitis seems more important than ruling in. If antibiotic treatment is considered, complicated appendicitis should be ruled out. Therefore, sensitivity and NPV for complicated appendicitis must be high. Ruling in complicated appendicitis is less important, as patients with a false positive test for complicated appendicitis—actually having uncomplicated appendicitis—will undergo surgery, which is at the moment the standard therapy for any acute appendicitis.

## Diagnostic Work-Up for Acute Appendicitis

Several guidelines, international and national, give advice about the diagnostic work-up for suspected acute appendicitis (2,3). The diagnostic work-up differs per country, which leads explicitly to differences in NARs (14). Some guidelines use scoring systems, some only use clinical assessment of the treating physicians, and some guidelines use standardized imaging to diagnose acute appendicitis in all patients or in a selected group of patients (2,3,5,15).

### Clinical View

The traditional way of setting a diagnosis is based on clinical assessment. History taking and physical examination combined with laboratory findings are still seen as the cornerstone of diagnosing acute appendicitis, but have a high intra-observer variability and a far from perfect accuracy. The correct clinical diagnosis of both surgical trainees and surgeons failed in 44% and 43% of patients with acute abdominal pain, respectively, when based on medical history, physical examination findings, and routine laboratory tests, but no imaging (16). For the diagnosis of acute appendicitis, diagnostic accuracy of trainees and surgeons is comparable; sensitivity and specificity vary from 76% to 85% and 82% to 87%, respectively (16). These data mean that if only the clinical assessment of the surgeon or surgical trainee is used to set a diagnosis in patients with suspected appendicitis, 15%–24% of patients with acute appendicitis are missed and an NAR of 13%–18% will be seen, which is much higher than the ideal upper limit of 5%. Therefore, patients cannot be accurately ruled in or ruled out based on clinical assessment only.

### Laboratory Tests

In addition to clinical examination, laboratory tests such as white blood cell (WBC) count or C-reactive protein (CRP) are widely used as a next step in diagnosing acute appendicitis (3). Individually, these inflammatory markers are weak discriminators, but when combined they achieve a higher discriminatory power in diagnosing acute appendicitis versus no appendicitis (17). Nevertheless, according to a study of prospective data, including 1024 patients presenting with clinical suspicion of acute appendicitis, this combination is not able to sufficiently rule in or rule out appendicitis (18). By exploration of different cut-off values for WBCs and CRP, a maximal NPV of 88% can be reached for ruling out appendicitis (18). Although this maximal NPV seems high, it is found only in a small subgroup (9.9% of the total cohort) consisting of typical patients having WBC <10 × 10^9^/L or CRP <10 mg/L. Since the NPV is less optimal in other patient categories, ruling out appendicitis based only on laboratory tests is not a good strategy. For ruling in appendicitis based on inflammatory markers only, a PPV up to 74.2% is found; this maximum is seen in patients with WBC >20 × 10^9^/L who comprise only 6.1% of the 1024 patients in the cohort (18). Therefore, ruling in appendicitis based only on laboratory tests is even more troublesome than ruling out. This is not surprising as CRP and WBC are general and non-specific inflammatory markers, and thereby less helpful for a specific diagnosis. Structured models, such as clinical scoring systems including laboratory tests, may be helpful.

### Clinical Scoring Systems

To overcome subjective interpretation of clinical signs and lab tests, standardized clinical risk scores have been composed. Originally, the Alvarado score is the best-known scoring system based on clinical parameters for the diagnosis of acute appendicitis. However, standards have changed to more recent scoring models. The 2020 update of WSES Jerusalem guidelines for diagnosis and treatment of acute appendicitis recommend the use of the Appendicitis Inflammatory Response Score (AIRS) and the Adult Appendicitis Score (AAS) as diagnostic scores of acute appendicitis (3), see Table 1.

**Table 1:** Clinical diagnostic scores: Alvarado, AIRS, and AAS.

		Alvarado	AIRS	AAS
Migration/relocation of pain		1	–	2
Anorexia		1	–	–
Nausea/vomiting		1	–	–
Vomiting		–	1	–
Pain in RIF/RLQ		2	1	2
Rebound pain/tenderness:	Mild	1	1	–
	Moderate	1	2	–
	Severe	1	3	–
Guarding:	Mild	–	–	2
	Moderate/severe	–	–	4
RLQ tenderness:	Women (16–49 years)	–	–	1
	All other patients	–	–	3
Elevated temperature		1 (>37.5 °C)	1 (>38.5 °C)	–
WBC (×10^9^)	7.2–10	–	–	1
	10–10.9	2	1	1
	10.9–14	2	1	2
	14–15	2	1	3
	⩾15	2	2	3
Shift of WBC to the left (>75% neutrophils)		1	–	–
Polymorphonuclear leukocytes		–	0 (<70%)	2 (62%–75%)
			1 (70%–84%)	3 (75%–83%)
			2 (⩾85%)	4 (⩾83%)
CRP level, mg/L for symptoms <24 h		–	0 (<10)	2 (4–11)
			1 (10–49)	3 (11–25)
			2 (⩾50)	5 (25–83)
				1 (⩾83)
CRP level, mg/L for symptoms >24 h		–	0 (<10)	0 (<12)
			1 (10–49)	2 (12–152)
			2 (⩾50)	1 (⩾152)

AAS: adult appendicitis score; AIRS: appendicitis inflammatory response score; CRP: C-reactive protein; RIF: right iliac fossa; RLQ: right lower abdominal quadrant; WBC: white blood cell.

#### Low-risk for appendicitis (rule out)

A diagnostic score may be able to classify a subgroup of patients as “low-risk for acute appendicitis,” which is used to rule out acute appendicitis. A recent study has validated 15 scoring systems for the identification of these low-risk patients in a cohort of patients presenting with acute right iliac fossa (RIF) pain in the United Kingdom (19). According to their standards, the ideal scoring system has a high specificity, while maintaining a failure rate (1-NPV) of less than 5% (19). In other words, an ideal appendicitis score should be able to (1) correctly classify patients without appendicitis as “no appendicitis” or rule out acute appendicitis accurately (in terms of a maximum acceptable failure rate of 5%) and (2) correctly classify patients with appendicitis as “appendicitis” in terms of the best achievable specificity. After finding the best model, the scoring systems have been externally validated in data sets from other European countries (19). In the British cohort of 3613 women, the AAS performs best. Using a cut-off score of 8 or less, a specificity of 63.1% is found, associated with a failure rate of only 3.7%. This means that based on the AAS, only 69 out of 1856 patients who score a low-risk of acute appendicitis do have an appendicitis. However, external validation in other countries has resulted in a failure rate up to 17.5%. In 1732 male British patients, not the AAS but the AIRS has performed best; the failure rate was 2.4% with a specificity of 24.7% at a cut-off score of 2 or less. This failure rate, however, increases during validation in cohorts from other countries, and is as high as 32%. The RIFT study group states that their study results “should be extrapolated cautiously to settings outside the UK” (19). Indeed, although the results of the RIFT study group seem promising in a cohort of UK patients, ruling out appendicitis based on a mere clinical scoring system does not perform well in other cohorts and will also not enable differentiation between complicated and uncomplicated appendicitis (19).

#### High-risk for appendicitis (rule in)

Ruling in appendicitis is a different story; these patients are classified as “high-risk for acute appendicitis.” The WSES guideline suggests that cross-sectional imaging may be avoided in patients younger than 40 years with a high-risk for appendicitis score result and one can proceed to (diagnostic) laparoscopy. High-risk patients are defined as patients with an AIR or Alvarado score of 9 and higher, or an AAS of 16 and higher. However, meta-analysis data on ruling in acute appendicitis based on clinical risk scores are lacking. Several studies have validated scoring models for the identification of patients at high-risk for appendicitis. Four studies have validated the Alvarado score (20–23), five the AIRS (21,23,20,24,25), and two the AAS (23,26), but results are very heterogeneous. We used a bivariate logit-normal random-effect model to pool results of the included studies for present review; the reported sensitivity and specificity for the Alvarado score were 24% and 97%, for the AIRS 22% and 97%, and for the AAS 53% and 93%, respectively. With a median prevalence of acute appendicitis of 37%, this would lead to a PPV of 82% for the Alvarado score, 81% for AIRS, and 82% for AAS. Although a high specificity is reached, these PPVs will lead to high NARs (18%–19%) when the final diagnosis of acute appendicitis is made only based on one of these clinical diagnostic rules. Standard use of imaging leads to less negative appendectomies (14) and therefore lower avoidable risks for the patient.

#### The three test zone concept (low-, intermediate-, and high-risk scores)

The concept of three test zones with two cut-off points for appendicitis scores to determine the need for imaging to diagnose appendicitis seems promising (27). However, we need more reproducible data in different cohorts, showing a stable performance of such test zones, before such a clinical decision rule can be used to bypass imaging. Even if patients can be pointed out being at “high risk” of having acute appendicitis based on a three-zone clinical score, a low-dose computed tomography (CT) scan (or step-wise conditional CT after initial ultrasound (US)) seems less harmful than standard “diagnostic” surgery. Surgery without imaging is accompanied by higher NARs compared to the known rates after a standard imaging approach (14,28). This argument is illustrated by comparing two prospective national SNAPSHOT audits (14). Standard imaging, by means of the step-up approach of US with additional CT or magnetic resonance imaging (MRI) if needed, in 1934 Dutch patients of whom 99.5% underwent preoperative imaging, resulted in an NAR of 3.2% (14). Within a population of 3326 British patients, only 32.8% underwent preoperative imaging, which resulted in a NAR of 20.6% (14).

### Imaging

To diagnose acute appendicitis with high accuracy, standardized imaging plays an important role. US, CT scanning, and MRI can all be used to diagnose acute appendicitis. With standard US equipment, the appendix can be visualized using a graded compression technique. Contrast-enhanced abdominal CT can be performed in the portal-venous phase (15). Studies show that intravenous contrast enhanced low-dose CT has comparable accuracy to normal-dose CT and should therefore be preferred (28,29). CT protocols are mainly based on helical scanners with a single detector or multidetector and slice thickness of 3–5 mm with an interval of 3–10 mm (30,31). Alternatively, MRI can be performed. Standard is the use of a 1.5T MRI with half-Fourier-acquisition single-shot turbo spin-echo (HASTE) sequences and a combination of T1, T2, and T2 fat suppression (15,32). Intravenous contrast can be used in MRI, but is not standard (15,32).

While US usually is low-priced, quick, and has no burden on ionizing radiation, CT and MRI reach better diagnostic results. According to a meta-analysis by Giljaca et al. (33), US alone has a sensitivity and specificity for acute appendicitis of 69% and 81%, respectively. Meta-analyses of Van Randen et al. (30) and Duke et al. (32) demonstrate that CT and MRI are better in detecting acute appendicitis than US with a summary sensitivity and specificity for acute appendicitis of 91% and 90% for CT, and 97% and 96% for MRI. The median prevalence of US, CT, and MRI, as reported in the reviews, is 76%, 50%, and 58%, respectively. Since a pre-selection probably resulted in higher prevalence of acute appendicitis, results of these imaging studies cannot directly be compared to those of the clinical diagnostic scores. For practicable test characteristics as PPV and NPV, this prevalence is essential. For instance, suppose that a highly specific test is applied in an unselected population with low prevalence of appendicitis. This results in low PPV, but high NPV. Conversely, within a selected high-risk group of patients, a low NPV and a high PPV could be found. When incorporating the mentioned prevalences into the calculations, PPV and NPV for US were 92% and 45%, respectively, for CT 90% and 91%, and for MRI 97% and 96%. If the before mentioned clinical scores would be applied to a population of patients suspected for appendicitis with an appendicitis prevalence of 50%, similar to the prevalence in the CT study population, this would result in a PPV and NPV of 89% and 56% for the Alvarado score, 88% and 55% for the AIRS, and 88% and 66% for the AAS. The diagnostic characteristics of CT and MRI are therefore much better than achieved by the three clinical diagnostic scores.

A more pragmatic approach instead of using only one imaging modality, may be using a diagnostic work-up in which an initial US is performed, followed by a conditional CT or MRI in case of an inconclusive or negative US (34). Leeuwenburgh et al. have demonstrated that the combination of US and CT leads to a sensitivity and specificity of 97% and 91% for the diagnosis of appendicitis. At the published study prevalence of 51%, a PPV of 92% and an NPV of 97% were found for conditional CT strategy. For US with conditional MRI, sensitivity, specificity, PPV, and NPV are 98%, 88%, 88%, and 98%, respectively (34). However, there are some limitations. US has high inter-observer variability, which leads to a diagnostic accuracy that varies between different radiologists. For CT, radiation and intravenous contrast are used. Especially in fertile females, children and young adults, this should be avoided if possible. However, low-dose CT has comparable accuracy (28,29,35), markedly reducing the possibility of radiation-induced cancer. Contrast allergy and contrast-induced nephropathy are infrequent. Experience in reading an MRI is less common among radiologists and some may need additional training (36). However, training with direct feedback improves the accuracy of both radiologists and residents even after evaluating only 100 cases, with sensitivity and specificity reaching 92% and 88%, respectively, for the diagnosis of appendicitis (36). In addition, MRI has a longer in-room time, is logistically challenging, and may not be available 24/7. Costs of diagnostics are also important. Although imaging has higher initial costs, standard imaging has shown to be cost-effective (37) as well as reducing the NAR (14).

## Discriminating Complicated from Uncomplicated Appendicitis

Guidelines do not clearly advise how to differentiate between uncomplicated and complicated appendicitis (2,3). Nevertheless, the same guidelines state that complicated appendicitis should be treated within higher urgency, and that uncomplicated appendicitis may be treated with antibiotics only (2,3). Due to these different strategies, differentiation between uncomplicated and complicated appendicitis has become more relevant. In order to differentiate treatment according to severity of appendicitis, we need to establish uniform criteria for findings suggestive of complicated appendicitis and determine factors that are predictive for failure of conservative treatment in patients who were initially diagnosed with uncomplicated appendicitis. As mentioned in a previous section, the main purpose is ruling out complicated appendicitis.

While many studies have analyzed the diagnosis of acute appendicitis itself, only a few have tried to distinguish between uncomplicated and complicated appendicitis. Several studies have described the capability of the AIRS and Alvarado score to discriminate uncomplicated from complicated appendicitis (38–41). None of these studies mentioned diagnostic accuracy measures, and therefore sensitivity and specificity cannot be calculated. Two other studies reported the design of a scoring system including clinical and biochemical features; neither reported diagnostic accuracy measures (42,43). Imaging seems to be an essential step in differentiating uncomplicated from complicated appendicitis. A recent meta-analysis identifies CT features such as abscess, extraluminal air, intra- and extraluminal appendicolith, and periappendicular fluid to be associated with complicated acute appendicitis (44). Although high specificity is reached, all parameters fall short in sensitivity (44), and are therefore not able to reliably rule out complicated appendicitis. Appendicolith on imaging as risk factor for complicated appendicitis is discussed below.

Only two studies have described a scoring system combining clinical and imaging features to distinguish between uncomplicated and complicated appendicitis, see Table 2 (45,46). Atema et al. (45) have developed two Scoring systems for Appendicitis Severity (SAS) that combine imaging with clinical and biochemical features: one using US features (SAS-US) and the other using CT features (SAS-CT). Sensitivity, specificity, PPV, and NPV for US-SAS are 97%, 46%, 42%, and 97%, respectively (45). For the scoring system with CT features, SAS-CT, these test features are 90% sensitivity, 70% specificity, 55% PPV, and 95% NPV (45). The SAS scoring systems provide excellent diagnostic characteristics (high sensitivity and NPV) to rule out complicated appendicitis, but do not perform well in ruling in complicated appendicitis. Avanesov et al. (46) have also developed a scoring system, the APpendicitis Severity Index (APSI), that combines with clinical and biochemical features with CT features. Sensitivity, specificity, PPV, and NPV were 82%, 93%, 92%, and 83% (46). That scoring system provides diagnostic characteristics (high specificity and PPV) needed for accurate ruling in of complicated appendicitis. The major drawback of these three scoring systems is that none have been validated externally in prospective studies yet.

**Table 2:** Appendicitis severity scores.

		SAS-US (45)	SAS-CT (45)	APSI (46)
Age	<45 years	0	0	0
	⩾45 years	2	2	0
	⩾52 years	2	2	1
Body temperature (°C)	⩽37.0	0	0	0
	37.1–37.4	2	2	0
	37.5–37.9	2	2	1
	⩾38.0	4	4	1
Duration of symptoms ⩾48 h		2	2	1
WBC count >13 × 10^9^/L		2	2	–
C-reactive protein (mg/L)	⩽50	0	0	–
	50–100	4	2	
	>100	5	3	
Imaging parameters, based on US (SAS-US) or CT (SAS-CT and APSI)				
Appendiceal diameter	⩾14 mm	–	–	1
Periappendiceal fluid		2	2	2
Extraluminal air present		–	5	1
Appendicolith		2	2	–
Abscess		–	-	3

APSI: APpendicitis Severity Index; CT: computed tomography; SAS: Scoring systems for Appendicitis Severity; US: ultrasound; WBC: white blood cell.

A recent systematic review and meta-analysis from our group compared all available studies on imaging modalities differentiating between uncomplicated and complicated appendicitis (47). Eleven studies using CT were found. Summary estimates were 78% for sensitivity and 91% for specificity, resulting in a PPV of 74% and an NPV of 93% for diagnosis of complicated appendicitis (47). Results were highly heterogeneous, with sensitivities ranging from 28% to 95% (47). One study has described the discriminatory capability when an initial US is performed followed by a conditional CT or MRI in case of an inconclusive or negative US. A sensitivity of 48%, specificity of 93%, PPV of 68%, and NPV of 84% are found for the diagnosis of complicated appendicitis (48).

Randomized trials comparing conservative and surgical treatment of uncomplicated appendicitis have found remarkable differences in number of erroneously included patients with complicated appendicitis. Two large randomized control trials (RCTs) have used standardized CT in their diagnostic approach to select only patients with uncomplicated appendicitis for study inclusion (49,50). While both trials used a CT protocol with standard intravenous contrast, Vons et al. (50) found complicated appendicitis in 18% of patients randomized for surgery versus only 1.5% in the surgery group of Salminen et al. (49). This difference may be explained by the fact that Salminen et al. (49) exclude patients presenting with an appendicolith before randomization. Post hoc analyses of Vons et al. (50) show a significant association between the presence of an appendicolith and the diagnosis of complicated appendicitis. In fact, when excluding the subgroup of patients without the presence of an appendicolith, there is no difference in 30-day post-intervention peritonitis between operated and conservatively treated patients (50). The appendicitis acuta (APPAC) trial has excluded patients with an appendicolith before randomization, which may have led to the substantial lower percentage of unintentional included complicated appendicitis patients. The presence of an appendicolith has previously been described as a significant predictor of the need for surgery after failed conservative treatment in acute appendicitis (51), and this may be because of an association with complicated appendicitis. The most recent RCT on this subject, the Comparison of Outcomes of antibiotic Drugs and Appendectomy (CODA) trial, also demonstrates this association between the presence of an appendicolith and higher risk of complicated appendicitis in the included patients with an assumed uncomplicated appendicitis (7). In addition, a significant higher risk for appendectomy after initial antibiotic treatment is seen in patients with an appendicolith (7). Atema et al. (45) incorporated the presence of an appendicolith in their SAS scoring system to differentiate between uncomplicated and complicated appendicitis.

The previously mentioned meta-analysis by Kim et al. (44) found a pooled sensitivity and specificity of 43% and 74% of the presence of an intraluminal appendicolith for complicated appendicitis, and the presence of an appendicolith results in 2 points in both the SAS-CT and the SAS-US (45). Considering these numbers, an appendicolith does not seem to be decisive for differentiation between uncomplicated and complicated appendicitis. Nonetheless, the effect of excluding patients with an appendicolith in selection for antibiotic treatment appears to be significant on outcomes and appendicitis recurrence rates in large RCTs (7,49,50), and therefore, it does have clinical impact and in further studies, better defining the role of appendicoliths is needed.

## Conclusion

Although the subject of appendicitis diagnostics is not new, a watertight work-up to accurately diagnose acute appendicitis remains challenging. A two-stage diagnostic work-up with adequate accuracy in both steps is needed. In the first diagnostic stage, acute appendicitis must be distinguished from other urgent or non-urgent abdominal disease diagnoses. In the second diagnostic stage of patients diagnosed with acute appendicitis, a differentiation between complicated and uncomplicated appendicitis is needed.

As no clinical or laboratory test has both high sensitivity and high specificity, relying only on such parameters means balancing the tradeoffs between the risk of delaying treatment of complicated appendicitis (inadequate sensitivity for complicated appendicitis) and the risk of negative surgical explorations (inadequate specificity for complicated appendicitis). Standard imaging increases the diagnostic power for both ruling in and ruling out appendicitis. Imaging can be combined with or even incorporated in scoring systems. Moreover, imaging plays an important role in differentiating between appendicitis and other abdominal pathology, for those patients with abdominal pain suspected of a cause in need of treatment. Even if a clinical scoring model would be able to rule out acute appendicitis, imaging is still needed in most cases to correctly diagnose the cause of the abdominal pain for that particular patient. And in case of appendicitis, imaging is still needed for a differentiation between complicated and uncomplicated appendicitis.

Today, probably the most sensible way to use clinical scoring systems for suspected appendicitis is to select patients for immediate imaging (intermediate and high-risk scores) or reassessment the next day (low-risk scores). If a clinical diagnostic model stratifies a patient at low-risk of having acute appendicitis, reassessment the next day in outpatient setting or discharge to family physician care seems preferable over inpatient observation for adequate use of resources. Importantly, if all patients with a high-risk score for appendicitis based on clinical scoring systems undergo imaging, the NAR will be minimized to an acceptable level.

As non-operative management of uncomplicated appendicitis has been identified as a feasible and safe treatment option, cross-sectional imaging is obligatory to distinguish between uncomplicated and complicated appendicitis in the second stage of diagnosing acute appendicitis. Cross-sectional imaging can rule out complicated appendicitis to a certain extent, but when CT features are combined with clinical and laboratory features in the SAS scoring system, specifically designed to differentiate between uncomplicated and complicated disease among patients with acute appendicitis, NPV for complicated disease can be as high as 95%. Implementing standardized low-dose CT protocols for appendicitis diagnosis is of paramount importance to avoid unnecessary radiation in patients with suspected acute appendicitis. In addition, determination of uniform diagnostic predictors for complicated acute appendicitis or recurrent appendicitis after conservative treatment is essential in order to both adequately rule out complicated appendicitis and optimize patient selection for antibiotic treatment of uncomplicated appendicitis.

Identifying predictive factors for both non-responsiveness to antibiotic treatment after accurate diagnosis and recurrence after antibiotic treatment would lead to less appendicitis recurrences optimizing treatment outcomes. As the number of patients with uncomplicated acute appendicitis either not responding to antibiotic treatment or encountering appendicitis recurrence is quite low, we need international scientific collaboration combining large prospective patient databases to be able to identify these factors. Future trials need to investigate the potential further improvement of antibiotic treatment results with achieving optimal selection of patients with uncomplicated appendicitis.

Standardized and low threshold imaging plays an important role for accurate diagnosis of acute appendicitis. It reduces the risk that another diagnosis is missed as cause of abdominal pain in need of (urgent) treatment. It minimizes NARs and it may help to differentiate between uncomplicated and complicated appendicitis, which is important because this may lead to different management strategies. In addition, with innovations in diagnostic imaging modalities and CT equipment, the as-low-as-possible radiation principle without compromising diagnostic accuracy is improving rapidly. With the current and ever increasing improvements in CT techniques, especially so for the low-dose CT modalities, it is hard to imagine a diagnostic paradigm in acute appendicitis not taking advantage of modern imaging. In this respect, leaving out imaging features in scoring systems may have no promising future. A surgeon’s clinical assessment is and will always be needed as interpretation of results and act upon it remains a skill, but a surgeon needs to have the benefit from modern imaging, at least in middle- and high-income countries. Cross-sectional imaging is not needed in patients with abdominal pain at low-risk of appendicitis or any other disease requiring treatment.
